# “I thought cancer was one of those random things. I didn’t know cancer could be caught…”: Adolescent girls’ understandings and experiences of the HPV programme in the UK

**DOI:** 10.1016/j.vaccine.2011.03.101

**Published:** 2011-06-10

**Authors:** Shona Hilton, Emily Smith

**Affiliations:** MRC Social & Public Health Sciences Unit, 4 Lilybank Gardens, Glasgow G12 8RZ, United Kingdom

**Keywords:** Human papillomavirus, Teenagers, Perceptions

## Abstract

**Background:**

The UK human papillomavirus (HPV) vaccination programme aims to provide girls aged 12–13 with protection against two of the most carcinogenic strains (types 16 and 18) of this sexually transmitted virus which together account for 70% of cases of cervical cancer. Despite evidence suggesting a general lack of knowledge about HPV and its link with cervical cancer, vaccine uptake rates were generally high in the UK for the first year of the HPV vaccination programme. In countries that implemented the HPV programme ahead of the UK, studies have found that girls’ and parents’ levels of awareness about HPV have increased since implementation of the programme but that knowledge continues to be limited. This study offers some of the first insights from the UK into adolescent girls’ understandings of HPV, its link with cervical cancer, and experiences of vaccination, since the programme was introduced in September 2008.

**Method:**

Eighteen focus groups were conducted between December 2009 and May 2010 with schoolgirls aged between 12 and 18 living in various parts of the UK.

**Results:**

Eighty seven girls participated in these discussions. Typically, girls knew very little about HPV or how they could best protect themselves from HPV infection. Although many of the girls linked HPV to cancer, only half specifically associated it with cervical cancer. Most girls had no idea how long the vaccine would offer them protection. They assumed that HPV vaccination must be important for their health because it was recommended by people they trusted, namely parents and immunisation experts. Just over half of the girls were aware that in the future they would need to attend for cervical screening. Key concerns which girls expressed about HPV vaccination reflected their anxieties about needles, anticipated pain on injection, privacy during vaccination and fears about needle cleanliness.

**Conclusion:**

Our data point to a need to continue to address gaps in knowledge about HPV and to provide information to address girls’ concerns about vaccination. This could be achieved through targeted campaign materials and by ensuring those involved in delivering the programme are aware of girls’ anxieties to prevent limited knowledge and fears about vaccination becoming barriers either to HPV vaccination.

## 1 Introduction

In the UK a vaccination programme against the human papillomavirus (HPV) was introduced in September 2008. The programme aims to provide three doses of HPV vaccine to girls before they reach an age when the risk of HPV infection increases [1]. The programme currently offers girls aged 12–13 protection against two of the most carcinogenic strains of this sexually transmitted virus (types 16 and 18) which together are responsible for 70% of cases of cervical cancer [2]. A concurrent three year catch up programme is also being offered to girls aged 14–18 years. The latest uptake rates for all three doses are high among the younger cohort of girls (aged 12–13) in England 76.4% [3] and in Scotland 89% [4]. Uptake of all three doses among the oldest cohort targeted for the catch-up programme (17–18 years) has also been high in Scotland (85%), but lower uptake for these older age groups has been achieved in England (38.9%) [5].

These HPV vaccine uptake rates among the younger cohort of girls indicate high levels of acceptability of the HPV vaccine programme to date in the UK. This is despite the evidence of a general lack of knowledge among British women about HPV and its link with cervical cancer. For example, in a survey of 400 female university employees just 30% had heard of HPV and only 11% knew of its causal association with cervical cancer [6]. Similarly, in a survey of women attending a Well Woman clinic in London (UK) (*n* = 1032) about 30% recognised HPV only in name. On further questioning, less than half knew of the link with cervical cancer and there was confusion about whether condoms or oral contraceptives could prevent HPV infection [7]. Similarly, in a representative sample of British women (*n* = 1620) aged 16–97 years, a quarter of respondents were aware of HPV, and awareness was lower in those with less formal education [8]. Studies of parental attitudes to HPV vaccination have also found a lack of knowledge about HPV, but have nonetheless indicated support for the introduction of the vaccination programme [9,10]. Whilst it is important to note the high levels of support for the HPV vaccine despite limited knowledge of its role in the aetiology of cervical cancer, this balance could shift in the future. Studies of vaccine decision-making for younger children suggest that once a vaccine is perceived to have potential side effects, then gaps in knowledge, myths and misunderstandings about the diseases to be prevented can shift the balance of decision-making [11], since perceptions of the severity and likelihood of contracting the disease are a key factor considered in whether to accept a vaccine for younger children [12].

In recognition of the poor levels of knowledge about HPV, the public awareness campaigns were launched in the UK to accompany the introduction of the vaccination programme. Their launch coincided with intense media coverage of the diagnosis and death from cervical cancer of reality television star, Jade Goody. Whilst this media coverage might have been assumed to provide useful background information about cervical cancer and HPV in the lead up to the introduction of the new vaccination programme, an analysis of newsprint coverage of her illness and death found that it tended not to include factual or educational information that would help women to make connections between HPV, cervical cancer and the new programme [13].

Post-implementation studies continue to reveal limited public knowledge about HPV. A recent UK based interview study explored girls (aged 17–18 years) knowledge about HPV and attitudes towards HPV vaccination among girls who were part of the ‘catch-up’ vaccination programme. Ten interviews were carried out between March and May 2009. Williams et al.’s study found that most girls had limited understanding of HPV and HPV vaccination, and were uncertain about the need for the vaccine both in terms of perceived risk [14]. Similarly, a study of HPV knowledge following the implementation of the HPV vaccination programme in Australia found low levels of knowledge [15], and a US study conducted after publicity about the HPV vaccine produced by the manufacturers showed an increase in the perceived need for the vaccine, but no improvement in knowledge and understandings about why the vaccine was important [16]. In the UK public awareness about HPV after implementation of the vaccination programme still needs to be ascertained. This study therefore explores adolescent girls’ understandings of HPV and its link with cervical cancer, and their experiences of vaccination in the year following the introduction of the vaccination programme, in order to identify gaps in knowledge which could have important implications for future cervical cancer prevention in the UK.

## 2 Methods

### 2.1 Sampling and recruitment

Eighteen focus groups were conducted between December 2009 and May 2010 with schoolgirls aged between 12 and 18 years living in various parts of the UK. Purposive sampling was used to recruit a diverse sample in terms of socio-economic circumstances (see Table 1). Girls were recruited through posters, leaflets and adverts which were placed in a range of community settings including educational, community, and leisure and sport facilities. Adverts in local newspapers and strategically chosen websites, such as Facebook, Bebo, and Jo's Trust (a cervical cancer support website) invited interested parties to contact the researcher. Girls were also recruited through community group leaders such as Girl Guide leaders, community workers running youth groups in socially deprived areas, school teachers or parents who been contacted by the researchers or who had viewed an advert indicated they would be interested in getting their youth group, class or daughters involved. Each girl was given a £10 voucher for taking part.

### 2.2 Data collection and analysis

A topic guide, which was developed from the literature and pilot work, explored the following themes: knowledge and understandings about HPV infection and its link to cervical cancer; beliefs about safer sex and personal risk in relation to HPV; understandings and concerns about HPV vaccination; vaccination experiences; and understandings of the importance of cervical cancer screening. The group discussions were facilitated by ES and lasted between 1 and 2 h. All discussions were audio recorded (with participants’ permission) and transcribed verbatim. To enable systematic comparisons to be made across the large amounts of data, each transcript was checked and imported into NVivo 7. Data were thematically coded and systemically charted, following the principles of framework analysis [17]. One of the benefits of framework analysis is that it allows a team of researchers to rigorously examine and cross-compare data to identify common reasoning and themes, and ideas that are less common or specific to certain subgroups or individuals. Throughout the analysis attention was paid to any deviant or contradictory cases [18] and to group dynamics using the full transcripts supplemented by field-note observations [19].

### 2.3 Reporting data

To report the data we have selected quotes attributed to an individual which are expressed concisely and typify responses around key themes. We have also selected some extracts which convey the types of interactions which occurred in the group discussion to give a sense of the rich data gathered from group discussions, whilst being mindful of group effects and the fact that all conversation is influenced by the context in which it is generated [20]. An advantage of the focus group method is that it can generate dynamic data by encouraging discussion between group members [21]; however the chaotic nature of conversation in more animated groups can make it difficult to identify all the individual speakers and this was a particularly challenging aspect of this study.

Ethical approval for the study was obtained from the research ethics committee of the University of Glasgow's Law, Business and Social Sciences Faculty.

## 3 Results

Eighty seven girls took part in 18 focus group discussions, nine of which were conducted in Scotland (Strathclyde and Lothian regions) and nine in England (London region) (see Table 1). Participants were aged between 12 and 18 years of age. Seventy eight girls had been vaccinated against HPV, four had refused the HPV vaccination, and four had delayed vaccination as they were undecided; data were missing for one girl.

### 3.1 Understandings about HPV infection

Typically, participants knew very little about HPV infection and its transmission. They were asked if they knew how to protect themselves from HPV infection. Some girls mentioned the HPV vaccine, others mentioned that condoms would prevent transmission, or that avoiding sexual intercourse altogether would offer the best protection from contracting HPV. It was common for the girls who did know that HPV was sexually transmitted to believe that their own risk of contracting it was low because they associated HPV infection with girls who “sleep around” (FG S5: Noelle 13). Only two of the girls mentioned that they knew HPV infection is highly prevalent. Discussions about prevalence rates of HPV tended to lead onto conversations about whether HPV could be detected through routine STI testing. Although no routine test for HPV infection is available, it was common for girls to believe that boys were the vector of infection and should be routinely tested for HPV and given treatment if infected. This notion arose spontaneously in three groups. Further discussion revealed that girls were applying their general knowledge about STI prevention to HPV, although they were also unsure about whether HPV testing really was part of routine STI testing, as illustrated by the following extract from one group discussion:

**Sally**: Boys should be tested.
**Lorne**: But you won’t find HPV in an STI screening.
**Sally**: What do you mean?
**Lorne**: They won’t be looking for it.
**Sally**: Cervical cancer?
**Lorne**: They might look for something else. How's the clinic going to tell you that he's got cervical cancer?
**Sally**: I’m not sure. Can they?
**Facilitator**: No
(**FG E5: Sally 14, Lorne 16**)

This comment that boys could be screened for cervical cancer rather than HPV infection went unchallenged by the group members. This lack of a clear understanding of how HPV infection could be prevented and what the girls could do to protect themselves was particularly evident in the younger groups. For example, when one younger group was asked how they could protect themselves against HPV infection, they replied:

**Tess**: Take the pill.
**Joanne**: That's not going to help.
**Hannah**: The pill doesn’t help, does it, at all?
**Facilitator**: No
**Joanne**: That's pregnancy protection not health protection
(**FG E2: Tess 13, Joanne 13, Hannah 12**)

### 3.2 HPV and its link with cervical cancer

Around half of the girls were aware that HPV infection could lead to the development of cervical cancer, but there was also some confusion about whether cancer could actually be prevented. As one girl considered:

Cervical cancer. I thought it was just like any cancer, like kind of like lung cancer, it just kind of appears… like one minute you’re all right and the next minute it's like you’ve got cancer. I thought it was like that, I thought cancer was one of those random things. I didn’t know cancer could be caught like sexually transmitted at all **(FG S5: Lisa 15)**.

It was common for girls to discuss broader ideas about cancer and to mention a belief that cancer was difficult to control through any preventative measures. As one girl commented: “it doesn’t work in my brain that you can protect against cancer because cancer is meant to be one of those things that just happens and you can’t stop it” **(FG E8: Chloe 16)**. Although almost all of the girls were aware that Jade Goody had died from cancer many were unaware that she had had cervical cancer and few made any link to the HPV vaccination programme.

### 3.3 Understandings of HPV vaccination

It was common for the girls to mention having read the information leaflets about the HPV vaccination, but many reported that their mothers had been most instrumental in making the decision about whether HPV vaccination was in their best interest. Typically girls referred to the HPV vaccine as the ‘cancer jab’ but struggled to provide more specific detail about what the vaccine protects against. Girls within two groups knew that it protected against some form of cancer but were not sure precisely which cancers (FG S3, FG E4) Discussion in one group showed that they understood that the vaccine would not provide complete protection from all carcinogenic strains of HPV (FG E6), whilst another group believed the opposite to be true: “I think it protects you against all the types which cause cervical cancer” **(FG S11: Kelly 17)**. Girls in another group thought that the vaccine would stop them dying from but not getting cervical cancer.

“I think the vaccine, doesn’t prevent you from having cervical cancer. But it can, it stops you from getting it bad. You might not get the full dose of cancer, but you still get a small dose” **(FG E2: Tess 13)**.

Most girls had no idea how long the vaccine would provide protection against HPV, and one girl questioned whether the vaccine “might be a complete waste of time” **(FG S7: Lily 15)** given that it only protects against two HPV strains out of a huge number of possible strains. However, about a third of the girls did understand that the vaccine protected against the most carcinogenic strains.

When girls were asked about how they thought the vaccine worked and what the vaccine contained discussions tended to be short, full of pauses and tentative guesses. Few of the girls appeared to have given any thought to this prior to being asked in these group discussions. Among the few groups that did try to respond to this question there was a misunderstanding that the vaccine contained cancer cells. For example:

**Esther**: And do you know the injection is a bit of the cervical cancer?
**Kim**: That's what I just said, the dead virus
**Esther**: Oh sorry. But it's not dead, actually it's alive
**Jordan**: It's alive, no, for real, it's alive?
**Diane**: So you get three different doses
**Jordan**: Oh goodness
**(FG E1: Esther 13, Kim 12, Jordan 12, Diane 13)**

Despite such fears about the possibility of a live virus or live cancer cells being used in the vaccine, in general the safety of the vaccine was not a primary concern and there was little discussion of any long-term side-effects from the vaccine. There was also evidence of high levels of trust in the Government and immunisation experts that this vaccine must be good for their future health (otherwise it would not have been introduced). As Rose (FG 16) stated: “I think the people in charge, like Government's health people have decided the jag is in our interest so I feel there's no reason not to get it”. Thus the vaccine was generally assessed by the girls to be of low risk and high benefit to their health.

### 3.4 Understandings about future cervical cancer prevention

Just over half of the girls were aware of cervical smear tests. Most of these girls were also aware that in the future they would need to go for cervical smear tests themselves, although few knew at what age they would be first expected to attend for one. Most of the girls who knew about smear tests had learnt about them from their mothers, for example when their mothers had talked about receiving their own appointment cards for screening. It was also common for girls to recall that during their HPV vaccination school nurses had told them they would still need to go for smear tests in the future. Some girls had heard that smear tests were unpleasant but were aware of its necessity. This seemed most evident when they discussed Jade Goody's untimely death and several groups discussed the fact that she had missed attending for a smear test which led to the late discovery of her cancer (FGS- E7, E8, E9, S4, S7, S11), as illustrated by the following extract:

**Anna**: I think she [Goody] hadnae been for a smear or something.
**Beth**: But I would never no’ go for one, though… it would be quite embarrassing.
**Sheona**: You need to go.
**Lily**: Well if I didn’t go, I’d feel dead like guilty, like it would be like eating away at me. And then imagine if you didnae go for it and that happened? Like, that's quite bad… she could’ve stopped that a lot sooner.
**Sheona**: Especially like her when you’ve got children
**Olivia**: Like I don’t understand why she wouldn’t go if it was going to help her, I think she was a bit stupid.
**(FG S7: Anna 14, Beth 14, Sheona 15, Lily 15, Olivia 14)**

### 3.5 Experiences of vaccination

One of the issues that the girls seemed most keen to discuss was their experience of HPV vaccination. Whilst there were often silences and stilted conversation in discussion of their understandings about HPV infection and its prevention, conversation was animated and the girls frequently interrupted or spoke over each other when recalling their experiences of receiving the vaccination. This was particularly evident in relation to their fear of needles and the pain of injection, the issue of privacy during vaccination, and concerns about needle cleanliness.

Across the focus groups, it was common for girls to discuss feeling scared about getting the vaccine and worried about the level of pain caused by the needle. This was discussed in all of the groups and ranged from girls describing a mild sense of nervousness, to feeling tearful or sick with anxiety. In four groups girls talked hypothetically about refusing the HPV vaccine due to what they described as ‘needle phobias’ but only one girl actually stated that she had refused the vaccine because of a needle phobia. Girls frequently described difficulty controlling a range of emotions in front of class mates. As one girl described:

“We were all standing waiting and the fear was building. Me and my friend were crying coz we didn’t want to get it. People were laughing at us. It weren’t funny. And afterwards, we saw them crying, so we were laughing then” **(FG E3: Fran 14)**.

In almost all of the groups there was also discussion of various myths and rumours circulating about the vaccination. These seemed to stem from the fact that three doses of the vaccine were required, and the prospect of three injections often became more daunting as rumours spread. Typical rumours were that each injection was more painful than the previous one, that the needle became larger with each dose, or that the dose became “thicker” and “larger”. For example, when one girl said: “I’ve been told the third's the worst” **(FG S4: Clara 16)** the other group members nodded and one replied that she had heard that the third injection was “double strength and like a bigger needle” **(FG S4: Millie 16)**. Although these rumours often appeared to have come from other girls, there were also examples of rumours spread by boys, particularly in relation to the site of the vaccination: “… they [the boys] said that we’d get it in your bum in your cervix” **(FG E2: Joanne 13)**. Whilst some girls found these stories worrying others dismissed them. One of the greatest concerns that the girls mentioned was their fear of needles. This was often of far more immediate concern when they were weighing up the pros and cons of vaccination than the possibility of future cervical cancer. This was succinctly summarised by one girl who said: “Teenagers, like now, you don’t think you’re going to get cancer so it's not important, and you think there's a needle – oh my gosh, I’m not going to get this. I’m scared of injections, so you don’t think about the long term, like it's going to be really useful” **(FG S4: Bella 16)**.

Another issue that arose in some groups was the issue of privacy. Typically, the girls described getting the vaccine in the school hall or a classroom with partitions which they saw as inadequate. As one girl recalled: “It wasn’t very private or anything. It was like, there was a like a pin board and then you behind, not very private, especially with the first one when you’re a bit worried **(FG S2: Sharron 13)**. Other girls recalled having forgotten to wear a vest top and being concerned about having to remove their school shirts to receive the vaccine. One girl said: “some folk were quite embarrassed about ‘cause like if you’ve got a long sleeved shirt on, which most of us did have, cause we wear white shirts, then you had to actually take their shirt off to get the jag, cause you couldn’t roll your sleeves up” **(FG S8: Megan 16)**.

The issue of needle cleanliness arose spontaneously in a few groups and was discussed at length in one group which debated whether they could trust that the health professionals would do the vaccinations in a way which meant that the needles were not accidentally re-sheathed and re-used. Some girls mentioned that the nurses seemed harassed, and the ‘conveyor belt’ method of delivery raised concerns about cross infection. A few girls described feeling anxious at seeing batches of syringes and needles lying on tables, as illustrated below:

**Annie**: To be honest, I’m not even sure if it's [the needle] clean
**Izzy**: No, I watched her, the nurse to made sure she took a new needle
**Michelle**: I know my doctor's is clean – I’m not sure about the school. You never know if the cleaners came in that day and they put the things for vaccination on the dirty table. Not clean at all.
**(FG E9: Annie 15, Izzy 15, Michelle 15)**

When girls were asked whether they had been given information or the opportunity to allay these concerns, most said they had not.

## 4 Discussion and conclusion

Our findings support those from a similar study by Williams and colleagues which used individual interviews to elicit understandings of adolescent girls post HPV vaccination implementation [14]. Consistent with this study, we found that girls knew very little about HPV prevalence and transmission. These findings also echo earlier research on older women's knowledge about HPV prior to the introduction of the HPV vaccination programme [7–10]. The lack of knowledge about HPV prevalence and its transmission is of concern because it may impact on future health behaviours. Our data suggest that HPV prevalence is underestimated and that as a result girls assess their own likelihood of contracting HPV as low, believing that HPV infection was only common among people who had multiple sexual partners. This notion may have arisen from media reporting about HPV and the development of the vaccine; some media coverage reported concerns that HPV vaccination might increase the risk of promiscuity among adolescents [22], whilst little news coverage reported that HPV is a highly infectious and prevalent virus within the general population, or that around 20% of girls will have contracted HPV by the time they reach 18 years of age [23]. Waller and colleagues have argued that an emphasis on the high prevalence of HPV in the population may be useful in helping to increase the acceptability of HPV vaccination if people perceive the likelihood of contracting HPV infection to be high [24].

In contrast to concerns that in targeting of HPV campaign material at sexually active young women, girls could be presumed to be the source of HPV infection [25], our study found that some girls viewed boys as the vector of infection. Indeed there was much discussion among participants about the need for boys to be tested routinely for HPV as part of STI screening and treated if infection was detected. This demonstrates how in the event of not knowing about HPV infection, participants tended to draw on their other knowledge about sexually transmitted infections such as chlamydia. It also highlights the level of confusion among some young people on what is a complex issue, which may have implications for how they assess the risks associated with HPV for their health. If girls assess that their own risk of contracting infection is low and that HPV infection could be amenable to treatment, vaccination could be deemed less important. Although HPV vaccine uptake is generally high, should uptake rates fall these data suggest that there is a need to make girls aware of the high prevalence of HPV and that their best form of protection is the vaccine. However, these misunderstandings could also have implications for the uptake of HPV should the programme be rolled out to include boys in the future

One limitation of this qualitative study is that the girl's self-selected into the study, and that despite advertising for girls who had not opted to have the HPV vaccine, we only managed to recruit eight unvaccinated girls. Nevertheless, this study does offer new insights about girls’ concerns and views on HPV and HPV vaccination which could be used as the basis to conducting a larger scale representative survey to identify which findings are generalisable. For example, we found a lack of any anxiety about long-term or serious side-effects from HPV vaccination and that these adolescent girls trusted their parents, the Government and immunisation specialists to make beneficial decisions and recommendations to protect their future health. It would be useful explore this finding to pinpoint when anxieties about vaccines start to occur and trust starts to erode. Roughly half of the girls were also aware that having the HPV vaccine did not negate the need to attend for cervical screening in the future; this message needs to be reinforced however for those girls who did not know this. Our research also suggests that whether girls attend for screening may be dependent on their own mother's participation in, and perceptions of the importance of, cervical screening. Another point worthy of addressing is that many girls believe that cancer is almost an inevitable part of life and questioned whether a vaccine could actually protect them against cervical cancer. This points to the need to continue to provide up-to-date information on how effective the HPV vaccine is estimated to be; if positive new data on HPV vaccine efficacy emerges this could be promoted through the media as a good news story in the battle against cancer [22].

Our study also suggests that it would be worthwhile addressing adolescents’ concerns about and the process of administering and receiving the vaccination, and to dispel myths surrounding HPV vaccination. Concerns about the cleanliness of needles, the size (of needles) and dose of the vaccine in the second and third doses and the extent of privacy that girls can expect whilst receiving the vaccine could be easily addressed through clear information, and it is important that these worries do not become barriers to a high uptake of immunisation.

In conclusion, our data provide some of the first insights from adolescent girls on HPV following the introduction of the UK HPV vaccination programme in 2008. Our data point to a need to continue to address gaps in knowledge about HPV and to provide information on girls’ immediate concerns about HPV vaccination. One method of doing this might be through targeted campaign materials and by ensuring those involved in delivering the programme are aware of girls’ anxieties so that girls’ limited knowledge and fears about vaccination do not act as barriers either to HPV vaccination.

## Figures and Tables

**Table 1:** Characteristics of focus groups and their participants.

Focus group	Recruited from	Pseudonym	Age	Had HPV vaccination?
1 (S1)	Affluent area: Strathclyde Snowballing in community, website	Lucy	14	Yes
		Emily	14	Yes
		Sarah	14	Yes
		Lynn	14	Yes
		Sammy	13	No
2 (S2)	Affluent area: Strathclyde Snowballing in community	Louise	13	Yes
		Sharron	13	Yes
		Mandy	13	Yes
		Eva	12	Yes
		Henretta	13	Yes
3 (S3)	Mixed area: Strathclyde Snowballing in community, website	Emma	13	Yes
		Megan	13	Yes
		Jane	13	Yes
		Elouise	13	Yes
		Julie	13	Yes
4 (S4)	Affluent area: Lothian School	Elaine	16	Yes
		Carla	16	Yes
		Fay	16	Yes
		Bella	16	Yes
		Kay	16	Yes
		Millie	16	Yes
5 (S5)	Mixed area: Strathclyde Community youth club	Kim	14	Yes
		Noelle	13	Yes
		Lisa	15	Yes
6 (S7)	Deprived area: Lothian School	Lily	15	Yes
		Nicola	14	Yes
		Gillian	14	Yes
		Olivia	14	Yes
		Sheona	15	Yes
		Nancy	15	Yes
		Amy	14	Yes
		Anna	14	Yes
		Jess	15	Yes
		Linsay	14	Yes
		Beth	14	Yes
7 (S8)	Deprived area: Lothian School	Megan	16	Yes
		Lottie	16	Yes
		Bethan	16	Yes
8 (S9)	Deprived area: Lothian School	Lia	18	Yes
		Sophie	17	Yes
		Ava	17	Yes
		Juliette	17	Yes
9 (S11)	Deprived area: Lothian School	Rachael	17	Yes
		Becky	17	Yes
		Sue	17	Yes
		Jill	17	Yes
		Katy	17	Yes
		Kelly	17	Yes
10 (E1)	Deprived area: London Community youth club	Jordan	12	Undecided
		Diane	13	Undecided
		Kim	12	Undecided
		Esther	13	Undecided
11 (E2)	Deprived area, London Community youth club	Tess	13	Yes
		Joanne	13	Yes
		Hannah	12	Yes
		Tanith	14	Yes
12 (E3)	Deprived area, London Community youth club	Natalie	14	No
		Fran	14	Yes
		Clara	14	No
13 (E4)	Deprived area, London Community youth club	Dawn	16	Yes
		Asha	16	Yes
14 (E5)	Deprived area, London Community youth club	Zora	Missed	missed
		Sally	14	No
		Lorne	16	Yes
		Cathy	17	Yes
		Claire	16	Yes
15 (E6)	Affluent area, Surrey Community youth club	Annabel	13	Yes
		Tess	13	Yes
		Rosie	13	Yes
		Vicky	13	Yes
		Shelly	13	Yes
16 (E7)	Affluent area: Surrey Community youth club	Rose	16	Yes
		Pat	15	Yes
		Lauren	15	Yes
		Matty	16	Yes
		Catherine	16	Yes
17 (E8)	Affluent area: Essex Community youth club	Chloe	16	Yes
		Cate	17	Yes
		Lorna	16	Yes
		Zoe	16	Yes
18 (E9)	Affluent area: Essex Community youth club	Fiona	15	Yes
		Izzy	15	Yes
		Isla	16	Yes
		Rhona	16	Yes
		Catrina	16	Yes
		Annie	15	Yes
		Michelle	15	Yes

*Groups prefaced with S were conducted in Scotland, and those prefaced with E were conducted in England.
